# Carbapenem-resistant *Enterobacteriaceae* bloodstream infections: A case-control study from a pediatric referral hospital in Argentina

**DOI:** 10.3389/fpubh.2022.983174

**Published:** 2022-08-25

**Authors:** Silvina Ruvinsky, Carla Voto, Macarena Roel, Verónica Deschutter, Daiana Ferraro, Norma Aquino, Vanesa Reijtman, María Eugenia Galvan, Eduardo Motto, Mauro García, Claudia Sarkis, Rosa Bologna

**Affiliations:** ^1^Coordinación de Investigación Clínica y Sanitaria, Hospital de Pediatría “Prof. Dr. Juan P. Garrahan, ” Ciudad Autónoma de Buenos Aires, Argentina; ^2^Servicio de Infectologia y Epidemiologia, Hospital de Pediatría “Prof. Dr. Juan P. Garrahan, ” Ciudad Autónoma de Buenos Aires, Argentina; ^3^Servicio de Microbiología, Hospital de Pediatría “Prof. Dr. Juan P. Garrahan, ” Ciudad Autónoma de Buenos Aires, Argentina; ^4^Servicio de Terapia Intensiva, Hospital de Pediatría “Prof. Dr. Juan P. Garrahan, ” Ciudad Autónoma de Buenos Aires, Argentina

**Keywords:** CRE-BSI, children, risk factors of acquisition, prognosis, use of resources

## Abstract

**Background:**

Antibiotic-resistant gram-negative bloodstream infections (BSI) remain a leading cause morbidity and mortality in pediatric patients with a high impact on the public health system. Data in resource-limited countries, including those in Latin America and the Caribbean region, are scarce. The aim of the study was to identify risk factors for acquiring carbapenem-resistant *Enterobacteriaceae* (CRE) bacteremia in children and to assess the use of resources.

**Methods:**

A retrospective case-control study was conducted to analyze demographic, epidemiological, clinical, microbiological, and outcome data as well as the use of resources between 2014 and 2019. Univariate and logistic regression analysis was performed in order to identify risk factors associated with CRE-BSI. The R software version 4.1.2 was used.

**Results:**

A total of 46 cases with CRE-BSI and 92 controls with gram-negative non-CRE-BSI were included. No statistical difference was observed regarding: median age (36 months; IQR, 11.2–117 vs. 48 months, IQR 13–119), male sex (50 vs. 60%), and underlying disease (98 vs. 91%) in cases vs. controls, respectively. The most frequent mechanism of CRE bacteremia were: KPC in 74%, OXA in 15%, and NDM in 6.5%. A total of 54.3% of cases vs. 32.6 % (*p* = 0.016) of controls were admitted to the pediatric intensive care unit (PICU), and 48 vs. 21% (*p* = 0.001) required mechanical ventilation. Bacteremia secondary to intra-abdominal infection was observed in 56.5% of cases vs. 35% of controls (*p* = 0.032). Previous colonization with CRE was detected in 76% of cases vs. 8% of controls. Combination antimicrobial treatment was most frequent in cases vs. control (100 vs. 56.5%). No difference was observed in median length of hospital stay (22 days; IQR, 19–31 in cases vs. 17.5 days; IQR, 10–31 in controls; *p* = 0.8). Overall case fatality ratio was 13 vs. 5.5%, respectively. The most statistically significant risk factors included previous PICU stay (OR, 4; 95%CI, 2–8), invasive procedures/surgery (OR, 3; 95%CI, 1–7), central venous catheter placement (OR, 6.5; 95%CI, 2–19), urinary catheter placement (OR, 9; 95%CI 4–20), mechanical ventilation (OR, 4; 95%CI, 2–10), liver transplantation (OR, 8; 95%CI, 2–26), meropenem treatment (OR, 8.4; 3.5–22.6) in univariate analysis. The logistic regression model used for multivariate analysis yielded significant differences for previous meropenem treatment (OR, 13; 95%CI, 3-77; *p* = 0.001), liver transplantation (OR, 13; 95%CI, 2.5–100; *p* = 0.006), and urinary catheter placement (OR, 9; 95%CI, 1.4–94; *p* = 0.03).

**Conclusion:**

CRE-BSI affects hospitalized children with underlying disease, mainly after liver transplantation, with previous urinary catheter use and receiving broad-spectrum antibiotics, leading to high PICU requirement and mortality. These risk factors will have to be taken into account in our region in order to establish adequate health policies and programs to improve antimicrobial stewardship.

## Introduction

Antibiotic-resistant gram-negative bloodstream infections (BSI) are one of the main healthcare-associated infections worldwide. Carbapenem-resistant *Enterobacteriaceae* (CRE) BSI are an emerging problem in children with significant morbidity, mortality and an important impact on public healthcare. BSI are more frequently nosocomial or healthcare related than community acquired (1).

The primary source of infection is not identifiable in around 30–50% of the cases (2–4). Most of the risk factors related to CRE-BSI are similar to those of bacteremia due to non-CRE gram-negative bacilli in general. Resistance to carbapenems can be acquired through the production of enzymes capable of hydrolyzing carbapenems or reduction of membrane permeability by loss of porins in combination with the production of extended-spectrum beta-lactamases or ampC beta lactamases (5). Invasive CRE infections occur primarily in pediatric patients with underlying conditions (6). According to information from the Argentine Antimicrobial Resistance Surveillance Program, carbapenem-resistant *Klebsiella pneumoniae* has increased in the general population from 16% in 2016 to 22.7% in 2019 (7, 8).

Therapeutic options for CRE-BSI in pediatrics are limited; therefore, the implementation of infection prevention and control measures and the identification of patients with risk factors for invasive CRE infections are a valuable tool in clinical practice.

Most information about risk factors for acquisition, clinical features, treatment options, prognosis, and use of resources is provided by studies in the adult population. Several studies have reported that risk factors for CRE-BSI in adults include critical illness, prolonged hospital stay, use of invasive devices, exposure to broad-spectrum antibiotics, and residence in nursing homes (9–12). Information from studies on CRE-BSI in children is scarce.

The aim of the study was to identify risk factors for acquiring CRE-BSI and clinical characteristics in children as well as the use of resources in a pediatric referral hospital in Argentina.

## Materials and methods

A retrospective case-control study covering a period between January 1, 2014, and December 31, 2019, was conducted at Hospital de Pediatría Prof. Dr. Juan P. Garrahan in Buenos Aires, Argentina. This hospital is a 534-bed, tertiary-care, pediatric center with special assistance for hematology-oncology patients, solid organ transplant recipients, and children with other underlying conditions. The age range of children included in the study was from 1 month to 18 years. Cases were defined as children with CRE-BSI confirmed by blood culture. Two control patients were selected for each confirmed case. Control cases were children with a confirmed infection with non-CRE-BSI. Cases and controls were selected consecutively from the microbiology lab database. The following data were collected for each patient: age, sex, epidemiological characteristics, underlying disease, comorbidities, clinical characteristics, microbiological features, treatment, use of resources (PICU admission, hospital length of stay), and final outcome. Data from the 3 months prior to infection regarding invasive procedures, PICU admission, broad-spectrum antibiotics use, and results of CRE colonization were also included in an electronic database.

The study protocol was approved by the Ethics Review Committee of the institution.

### Microbiological methods

The blood samples were inoculated in PF Plus® (for isolation of aerobic bacteria in pediatric patients) and FN plus® (for isolation of anaerobic bacteria) bottles and incubated in the automated Bact/Alert 3D® system for 5 and 7 days, respectively.

Bacterial identification was performed after 4 h of incubation of subcultures from positive blood culture broths using the MALDI TOF-MS (Vitek MS®). Sensitivity tests were performed using the Bauer-Kirby1 diffusion method and/or an automated system with the Vitek 2C® equipment. MIC to meropenem, sensitivity to fosfomycin, tigecycline, amikacin, and colistin were recorded. The interpretation of these tests was carried out following the recommendations of the Clinical and Laboratory Standards Institute guidelines (CLSI2).

Mechanisms of resistance to carbapenems were determined by phenotypic methods with colorimetric methods (Rapid CARB Blue Kit®) using subcultures of 4 h of incubation from positive blood culture broths and synergy tests with boronic acid (to detect KPC) and EDTA (to detect MBL) from the positive hemoculture broth after dilution in Mueller-Hinton broth. Molecular detection was performed using singleplex PCR for the *KPC* and *OXA* genes and multiplex PCR for *MBL* and was carried out in cases in whom determination of the type of *MBL* was necessary and to confirm *OXA* detection.

### Statistical analysis

For comparison of continuous variables, the Mann Whitney test and for categorical variables the Fisher's exact or Chi-square tests were used as appropriate. Univariate and logistic regression analysis was performed to identify risk factors associated with CRE-BSI. Logistic regression was used to explore the risk and protective factors for acquiring CRE-BSI in children. Variables that were significant in the F- test and Chi-square test at a level of *p* < 0.10 were included in the logistic regression model and clinical results from logistic regression are reported as odds ratio (OR), *p*-value, and 95% confidence interval (95%CI). The logistic regression model included all variables that retained significance after adjustment. Statistical significance was accepted at p < 0.05. Statistical analysis was performed using R software version 4.1.2.

## Results

A total of 46 cases with CRE-BSI and 92 controls with non-CRE-BSI were included in the study. Median age was 36 months (IQR, 11.2–117) in cases and 48 months (IQR, 12.8–119) in controls. No significant differences were observed related to age (*p* = 0.91) and sex (*p* = 0.28). Table 1 describes the demographic, epidemiological, and clinical characteristics of the cases and controls included. The presence of underlying conditions did not show a significant difference between groups (*p* = 0.15). Hematology-oncology disease was common in both the CRE-BSI and non-CRE-BSI groups; however, it was observed that liver transplantation was more frequent among cases than controls (*n* = 12; 26.1% vs. *n* = 4; 4.35%; *p* < 0.001). There were 96 vs. 54% hospital-acquired infections (*p* < 0.001) and 4 vs. 35% healthcare-associated infections in cases vs. controls, respectively. Median days from hospital admission to CRE-BSI was 35 days (IQR, 13.0–85), significantly longer than for non-CRE-BSI (11 days; IQR, 4–22.5; *p* = 0.023).

**Table 1:** Demographic and epidemiological data as comparative features between cases and controls.

**Variables**	**Cases *N* (%) *N* = 46**	**Controls N (%) *N* = 92**	**OR [95%CI]**	***p*-Value**
Age (months) [median (IQR)]	36 [11.2–117]	48 [12.8–119]		0.918**
Sex: female	23 (50)	37 (40.2)	1.49 [0.73–3.03]	0.282*
Underlying disease	45 (97.8)	84 (91.3)	4.29 [0.52–35.4]	0.157*
Type of underlying disease				
Hematology-oncology disease	23 (50)	46 (50)	1 [0.4–2]	1*
Liver transplant	12 (26.1)	4 (4.35)	7.76 [2.34–25.7]	<0.001*
Others (###)	1 (2.17)	0 (0)		
Associated morbidity (#)	6 (13)	14 (15.2)	0.84 [0.30–2.34]	0.754*
Colonization (##)	35 (76.1)	7 (7.61)	38.6 [13.8–108]	<0.001*
PICU (##)	26 (56.5)	23 (25)	3.90 [1.84–8.26]	<0.001*
Carbapenem treatment (##)	39 (85)	36 (39)	8.4 [3.5–22.6]	<0.001*
Surgery (##)	37 (80.4)	53 (57.6)	3.03 [1.31–6.99]	0.008*
Central venous catheter (##)	42 (91.3)	57 (62.0)	6.45 [2.13–19.5]	<0.001*
Mechanical ventilation (##)	22 (47.8)	16 (17.4)	4.35 [1.97–9.60]	<0.001*
Urinary catheter (##)	28 (60.9)	14 (15.2)	8.67 [3.81–19.7]	<0.001*

*Chi-square test.

^**^Kruskal-Wallis test.

#Associated morbidity: cases vs. controls: kidney failure, 8.7 vs. 6.5%; liver failure, 2 vs. 3%; and diabetes 2 vs. 3%; multiple organ failure, 2 vs. 0%.

##Three months previously.

###Other underlying diseases: cases vs. controls: structural abnormality of the gastrointestinal tract, 2.17 vs. 4.35%; liver disease, 4.35% in both groups; heart disease, 0 vs. 6.52%; neurologic disease, 2.17 vs. 4.35%; nephro-uropathy, 0 vs. 4.35%; bone marrow transplantation, 2.17% in both groups; genetic syndrome, 0 vs. 3.26%; short bowel syndrome, 0 vs. 3.26%; kidney transplantation, 4.35 vs. 0%; primary immunodeficiency, 2.17 vs. 1%; human immunodeficiency virus, 0 vs. 1%; prematurity, 2.17 vs. 0%; and other solid-organ transplantation, 2.17 vs. 0%.

**Figure 1:**
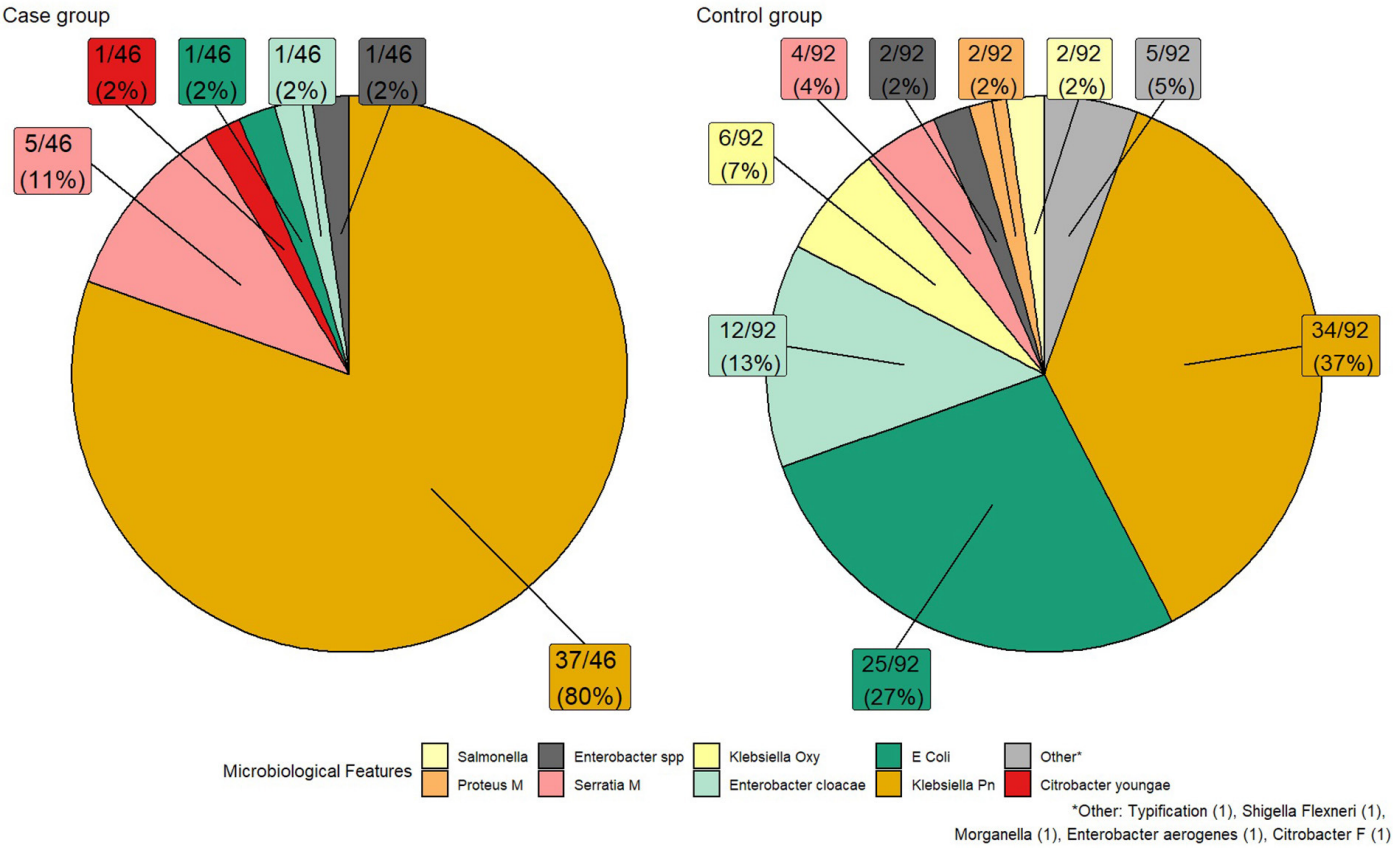
Bacterial distribution and resistance mechanism in cases vs control.

The microbiological isolates are described in Figure 1. The most commonly isolated *Enterobacteriaceae* among the cases were *Klebsiella pneumoniae* (80%) and *Serratia Marcescens* (11%) while among controls they were *Klebsiella pneumoniae* (39%), *Escherichia coli* (29%), and *Enterobacter cloacae* (14%). Antimicrobial susceptibility is presented in Figure 1. The most frequent resistance mechanisms among CRE-BSI group were KPC 74%, OXA 15%, and NDM 6.5%.

In the univariate analysis, several factors were associated with CRE-BSI (Table 1). Among patients with CRE-BSI compared to non-CSE-BSI patients, previous CRE colonization (OR, 38.6; 95%CI, 13.8–108; *p* < 0.001), a history of PICU admission in the last 3 months (OR, 3.90; 95%CI, 1.84–8.26; *p* < 0.001), exposure to Carbapenem (OR, 8.4; 95%CI, 3.5–22.5; *p* < 0.001), previous surgical procedure (OR, 3.03; 95%CI, 1.31–6.99; *p* = 0.008), central venous catheter use (OR, 6.45; 95%CI, 2.13–19.5; *p* < 0.001), mechanical ventilation (OR, 4.35; 95%CI, 1.97–9.60; *p* < 0.001); and urinary catheter use (OR, 8.67; 95%CI, 3.81–19.7; *p* < 0.001) were more likely.

In multivariate analysis, previous meropenem treatment (OR, 12.77; 95%CI, 3–77.28; *p* = 0.001), liver transplantation (OR, 12.81; 95%CI, 2.5–100; *p* = 0.006), and urinary catheter use (OR, 9; 95%CI, 1.38–94.22; *p* = 0.03) were independent risk factors for CRE- BSI.

### Main outcomes and use of resources

BSI were considered secondary (98% cases vs. 78% control; *p* < 0.001) to an intra-abdominal focus in 56.5% of CRE-BSI and 37% in non-CRE-BSI patients (*p* = 0.032). A clinical presentation with sepsis was common, without significant differences between the groups (*p* = 0.075). Clinical manifestations were similar in both groups (Table 2).

**Table 2:** Main outcomes and use of resources in cases and controls.

**Variables**	**Cases *N* (%) *N* = 46**	**Control *N* (%) *N* = 92**	**OR [95%CI]**	***p*-Value**
Sepsis	28 (60.9)	41 (44.6)	1.93 [0.94–3.98]	0.075*
Respiratory distress	9 (19.6)	8 (8.70)	2.55 [0.91–7.14]	0.082*
Kidney failure	5 (10.9)	8 (8.70)	1.28 [0.39–4.16]	0.680*
Neutropenia	22 (47.8)	24 (26.1)	2.60 [1.24–5.46]	0.013*
Combined treatment days (median [IQR])	17.5 [15.0–27.8]	10.0 [6.75–14.0]		<0.001**
PICU	25 (54.3%)	30 (32.6%)	2.46 [1.19–5.08]	0.016*
Mechanical ventilation	22 (47.8%)	19 (20.7%)	3.52 [1.63–7.59]	0.001*
Days on mechanical ventilation [median (IQR)]	18.0 [4.00–21.8]	9.00 [2.00–16.5]		0.488**
Inotropic drugs	19 (41.3%)	23 (25.0%)	2.11 [0.99–4.48]	0.056*
Length of PICU stay [median (IQR)]	16.0 [5.00–23.0]	8.00 [3.00–27.0]		0.506**
Length of hospitalization [median (IQR)]	22.5 [19.2–30.8]	17.5 [10.0–31.2]		0.819**

*Chi-square test.

^**^Kruskal-Wallis test.

Combination therapy was more common in cases than in controls (100 vs. 56.5%). The most frequent combination treatment in cases was meropenem and colistin (50%), meropenem, colistin, and tigecycline (24%), meropenem, colistin, fosfomycin, and tigecycline (9%), meropenem, colistin, and fosfomycin (4.5%), meropenem and tigecycline (4.35%), ceftazidime-avibactam associated with aminoglycoside (2%), and meropenem, colistin, fosfomycin, and tigecycline (2%). Prolonged meropenem infusion was administered in 37 (80.4%) cases and 7 (7.6%) controls.

Total antibiotic treatment was longer in CRE-BSI patients than in non-CRE-BSI patients (20 days; IQR, 16.0–31 vs. 10 days; IQR, 10–14.0; *p* < 0.001). Removal of the infectious focus was required in 51 vs. 17% in cases vs. controls, respectively (*p* < 0.001). Neutropenia was more frequently observed in CRE-BSI patients.

Patients with CRE-BSI were more likely to be admitted to the PICU and more often required mechanical ventilation than non-CRE-BSI patients (*p* = 0.001). There were no statistically significant differences in median length of hospital stay or median length of PICU stay (Table 2). The case fatality rate was 13% in CRE-BSI vs. 5.5% in non-CSE-BSI patients (*p* = 0.15).

## Discussion

In the last decade there has been a worldwide increase in invasive infections by multidrug-resistant gram-negative bacilli, particularly those with resistance to carbapenems, both in adults and in children (13, 14). A national survey conducted in Italy among participating centers of the Italian Association of Pediatric Hematology-Oncology recorded a two-fold increase in the CRE colonization rate and a four-fold increase in the incidence rate of CRE-BSI between 2012 and 2013 (15).

Our study was carried out at a pediatric tertiary-care center with assistance of children with severe underlying conditions, mainly patients with malignancies or solid-organ recipients. We observed that more than 90% of the patients in both groups had underlying diseases, being hematology and oncology diseases the most prevalent. In a cohort study conducted at our hospital, children with hematological disease and prolonged hospitalizations had a higher risk of bacteremia, with a predominance of gram-negative bacilli (16).

In CRE-BSI, the most commonly isolated organism was *Klebsiella pneumoniae*; all strains were tested for carbapenemase, and the most frequently found was KPC. These results are similar to reported national and international surveillance data (17–20).

Carbapenem resistance can be acquired by different mechanisms, including production of carbapenemase enzymes (KPC, OXA-48, MBL, NDM, VIM, and IMP) and production of an extended-spectrum betalactamase (ESBL) or AmpC-type betalactamase (21, 22) our control group, the resistance mechanism was extended spectrum beta-lactamase observed in 21%, similar to other reports in non-CRE-BSI (23).

Previous colonization with CRE was observed with a high percentage of the cases, similar to other series in children and adults (24). The means by which pediatric patients became colonized with CRE during antibiotic therapy were a long stay in wards with endemic CRE, handling by healthcare workers, or invasive procedures (25, 26). Colonization with multidrug-resistant *Enterobacteriaceae* has been described as a factor associated with the development of subsequent invasive infection. Akturk et al. found that 39% of children admitted to the ICU and colonized with KPC developed invasive infection (27). Our results showed a higher rate of previous CRE colonization among cases compared to controls. Actively screening CRE carriers should be considered in children at risk of CRE-BSI in order to implement isolation and control measures (28).

Previous invasive procedures (mechanical ventilation, surgery, urinary catheter placement), admission to the PICU, and broad spectrum antibiotics were significantly associated with CRE-BSI in our univariate analysis. These findings confirm previous reports showing these factors to increase the risk for acquiring CRE-BSI (29, 30).

In our study, we identified that previous exposure to meropenem, liver transplantation, and urinary catheter use were independent risk factors for the development of CRE-BSI. These findings are also consistent with previous publications (31). A retrospective cohort study of CRE-BSI patients documented that 50% had been exposed to carbapenems in the previous 6 months and that more than 90% had invasive devices and procedures at the time of bacteremia (31). In a US multicenter study, exposure to antibiotics with antipseudomonal activity in the past 3 months was found to be a significant risk factor for CRE-BSI colonization or infection (30). Logan et al. reported that children with CRE bacteremia had comorbidities, previous invasive procedures, and recent antibiotic exposure–particularly meropenem -, compared to those who were not infected (32).

Exposure to carbapenem in the 30 days prior to the infection was the only risk factor associated with carbapenem-resistant *Klebsiella pneumoniae* bacteremia in another series of children and adults with oncological and hematological disease (33).

Broad-spectrum antibiotics can modify the patient's normal gut flora by selection pressure increasing the risk of CRE. The implementation of antibiotic stewardship programs and active epidemiological surveillance programs are essential to avoid CRE hospital infections. In our institution we have an antibiotic stewardship program, which includes daily monitoring of medical prescriptions, timely identification of isolated microorganisms, antimicrobial sensitivity testing and appropriate initial treatment, as well as education programs for medical professionals of all areas in conjunction with the hospital's pharmacy (34).

In an observational study conducted in China, urinary catheter placement was an independent risk factor in children with nosocomial invasive CRE-BSI infections (35). A previous publication in adults showed that the presence of a urinary catheter increased the probability of invasive infections in patients with rectal CRE colonization (36).

Invasive procedures and medical devices may damage the mucosal barrier and provide a likely portal of entry in previously colonized patients.

CRE-BSI infections have been documented more frequently in children with underlying diseases and receiving immunosuppressive therapy (37, 38).

Phichaphop et al. found a high incidence rate of multi-resistant gram-negative bacilli infections in pediatric liver transplant recipients. In this cohort, 58.6% of isolations were ESBL-producing *Enterobacteriaceae* and 17.2% were CRE infections. An elevated Pediatric End-stage Liver Disease (PELD) score at the time of transplant was identified as an independent risk factor for the development of CRE-BSI during the post-transplant period (39). Pediatric liver transplant recipients are more frequently exposed to the hospital environment, invasive procedures, broad-spectrum antibiotic treatment, and immunosuppression.

In our patients with CRE-BSI, PICU admission was similar to those reported by Nabarro et al. however, no statistically significant differences were identified regarding length of hospital stay between the two groups (40). This finding might be explained by the fact that the control group was also exposed to a prolonged length of stay.

In our study, mortality was higher in CRE-BSI bacteremia than in non CRE bacteremia (13 vs. 5.5%), similar to other studies (6, 41). Nevertheless, CRE-BSI-related mortality was 13%, lower than in other published case series (40, 42).

Decreased survival has been observed in children infected with CRE, particularly those with hematology-oncology diseases and post-transplant patients receiving immunosuppressive treatment (37). This may be related to a delay in the indication of the appropriate empiric antibiotic as well as the patient's underlying condition.

Treatment of invasive CRE-BSI in children is not standardized. Observational studies suggest that combination therapy with at least two active drugs would have potential benefits in adult patients with invasive CRE-BSI (2, 43). Nabarro et al. documented a lower mortality rate in children with CRE-BSI who received a combination regimen with two active antibiotics (40).

One of the main limitations of our study is that it is a retrospective study conducted at a single referral center covering children with complex diseases; therefore, it may not be possible to extrapolate our results to those of other centers or other regions of the country. A large national controlled study is needed to better define risk factors, treatment, outcome, and mortality in children.

## Conclusion

CRE-BSI affects hospitalized children with underlying disease, mainly after liver transplantation, colonization with CRE, and receiving broad-spectrum antibiotics, leading to high PICU requirement and mortality. These risk factors will have to be taken into account in our region in order to establish adequate health policies and programs to improve antimicrobial stewardship.

## Data availability statement

The raw data supporting the conclusions of this article will be made available by the authors, without undue reservation.

## Ethics statement

The studies involving human participants were reviewed and approved by Comité Revisor y de Ética en la Investigación (CREI) - Hospital Prof. Dr. Juan P. Garrahan.

## Author contributions

SR and CV conceived the study. MR, VD, DF, NA, VR, MGal, EM, MGar, CS, SR, CV, and RB collected and analyzed the data. SR, CV, MR, NA, and VR drafted the manuscript. All authors contributed and approved the final manuscript.

## Conflict of interest

The authors declare that the research was conducted in the absence of any commercial or financial relationships that could be construed as a potential conflict of interest.

## Publisher's note

All claims expressed in this article are solely those of the authors and do not necessarily represent those of their affiliated organizations, or those of the publisher, the editors and the reviewers. Any product that may be evaluated in this article, or claim that may be made by its manufacturer, is not guaranteed or endorsed by the publisher.
